# The relationship between perceived peer support and academic adjustment among higher vocational college students: the chain mediating effects of academic hope and professional identity

**DOI:** 10.3389/fpsyg.2025.1534883

**Published:** 2025-02-21

**Authors:** Yaoxiu Zhu, Haidong Lu, Xinbo Wang, Wenling Ma, Min Xu

**Affiliations:** ^1^School of Psychology, Northeast Normal University, Changchun, China; ^2^Research Center of Mental Health Education in Weifang Business Vocational College, Zhucheng, China; ^3^China National Academy of Educational Sciences, Beijing, China; ^4^College of Chinese Language and Literature, Qufu Normal University, Qufu, China; ^5^School of Psychological and Educational Sciences, Zaozhuang University, Zaozhuang, China

**Keywords:** perceived peer support, academic adjustment, academic hope, professional identity, chain mediating effects

## Abstract

**Purpose:**

Academic adjustment is essential for the academic success of higher vocational college students. Although previous research has explored the role of perceived peer support in the academic adjustment of general undergraduate students, its role and underlying mechanisms in this group remain unclear. This study aims to explore the relationship between perceived peer support and academic adjustment, focusing on the chain mediating roles of academic hope and professional identity.

**Methods:**

A cross-sectional survey was conducted among 9,075 students from 35 higher vocational colleges across 15 provinces and cities in China, using multi-stage stratified random sampling. Validated scales were used to measure perceived peer support, academic adjustment, academic hope, and professional identity.

**Results:**

The findings revealed that perceived peer support not only directly influences academic adjustment but also exerts an indirect effect through the individual mediation of academic hope and professional identity, as well as through the sequential mediation from academic hope to professional identity.

**Conclusion:**

Perceived peer support, academic hope, and professional identity significantly promote academic adjustment among higher vocational college students. These findings provide theoretical insights into the mechanisms of academic adjustment and offer practical implications for educational strategies, emphasizing the importance of fostering perceived peer support, academic hope, and professional identity to enhance students’ academic success.

## 1 Introduction

Higher vocational education has gradually become a key force in promoting economic growth and technological innovation in economic globalization. As one of the world’s largest manufacturing powerhouses, higher vocational education in China is particularly prominent in terms of scale and influence. There are currently 1,560 higher vocational colleges in China, covering 16,000,000 students (China Daily HK, 2024). This large group of students (referred to as higher vocational college students) faces significant challenges in adapting to higher education, particularly with academic adjustment. Academic adjustment is a complex process involving students modifying their learning motivation, strategies, methods, and engagement in response to changes in their environment and learning needs, aiming to achieve psychological and behavioral balance (Baker and Sirky, 1986). Effective academic adjustment fosters academic success and enhances social and psychological adaptation. Conversely, students with inadequate academic adjustment frequently encounter various challenges, including declining academic performance, mental health issues, and difficulties in social integration (Van Rooij et al., 2018). Compared to their counterparts in general undergraduate programs, students in higher vocational colleges encounter distinct and complex challenges in academic adjustment, which need to be focused.

Firstly, higher vocational college students encounter distinctive learning environments and task requirements. During their transition from high school to higher vocational education, they must not only adapt to a new educational setting (Donaldson et al., 2023), but also assume new societal roles and responsibilities as adults (Cheung et al., 2020). Secondly, Many students enter higher education inadequately prepared for academic challenges, as evidenced by their insufficient foundational knowledge, inappropriate learning strategies, and poor time management skills (Pascarella et al., 2004). Thirdly, over 70% of these students in China are first-generation college students (Zhang et al., 2016), referring to students whose parents have the highest education level of high school or below (McFadden, 2016).

These students often face multiple challenges. On the one hand, they lack familial understanding and support for higher education (Jenkins et al., 2013), associated with lower levels of campus engagement and academic confidence (López et al., 2023). On the other hand, limited family and cultural capital further restricts their ability to access learning resources and developmental opportunities (Li et al., 2023). The cumulative effect of these factors not only exacerbates their academic adjustment difficulties but also impacts the quality of vocational education and their future career development. Consequently, conducting research on the academic adjustment of higher vocational college students can significantly enhance their academic performance and psychological well-being, while also providing a reference for improving the vocational education system.

Researchers have focused on the important roles of social support and psychological resources in academic adjustment. Perceived peer support, a major source of social support, refers to the help, care, and feedback that students receive from their peers or classmates in multiple dimensions, including emotional, informational, and material support (An and Guo, 2023). Previous research showed that perceived peer support could help reduce academic pressure and promote students’ academic adjustment by providing emotional support and learning resources (Worley et al., 2023; Zhang et al., 2021). In addition, academic hope and professional identity are the two key psychological resources. According to Hope Theory (Snyder et al., 2018), hope is an important psychological drive and cognitive process for individuals to achieve their goals. In educational contexts, academic hope refers to students’ expectations of academic goals and their confidence and motivation to achieve them (Sympson and Snyder, 1997). The professional identity reflects students’ deep cognitive and emotional identification with their major, which continues to develop throughout the learning process (Pifer and Baker, 2016; Qin, 2009). These two factors promote academic adjustment through different mechanisms: academic hope improves learning efficacy by stimulating goal-oriented learning strategies (Snyder et al., 2018), while professional identity promotes the academic adjustment by strengthening the sense of belonging and responsibility (Chu et al., 2021).

However, there is a relative lack of research on academic adjustment among higher vocational college students, particularly within the specific cultural context of China. Furthermore, existing research has been limited to a single theoretical framework, such as Social Support Theory or the Conservation of Resources (COR) theory, and a single-factor perspective. For example, previous research has examined the relationships between perceived peer support (Worley et al., 2023), academic hope (Wong and Yuen, 2023), and professional identity (Chu et al., 2021) and academic adjustment separately, however, the interaction mechanisms among these factors have not been fully elucidated. The limitations of this single perspective research impedes a systematic understanding of the mechanisms underlying academic adjustment among higher vocational college students, thereby constraining the development and implementation of effective support strategies.

**Figure 1:**
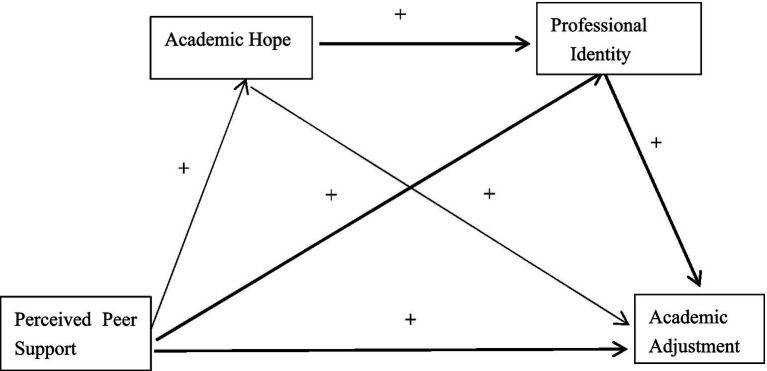
Theoretical research model of the study.

In summary, this study aims to explore the mechanisms of the academic adjustment of higher vocational college students by integrating perceived peer support, academic hope, and professional identity into a comprehensive theoretical framework. Additionally, it is expected to provide empirical evidence for educators to facilitate the development of more targeted support strategies, which is instrumental in promoting higher vocational college students’ academic success and holistic development. Furthermore, this study will provide a theoretical support for the improvement of vocational education policies. The theoretical model is shown in Figure 1.

### 1.1 The influence of perceived peer support on academic adjustment

Social Support Theory provides a robust theoretical framework for understanding the relationship between perceived peer support and academic adjustment. This theoretical perspective emphasizes that support received from social network plays a crucial role in stress management and environmental adaptation (Cohen and McKay, 2020). Within the higher education context, perceived peer support, as a significant component of social support, demonstrates substantial influence on students’ academic adjustment processes (Zhang et al., 2021). Perceived peer support facilitates academic adjustment through multiple mechanisms. Firstly, emotional support from peers can mitigate students’ anxiety and stress, provide psychological comfort, and enhance their capacity to address academic challenges (Camacho-Morles et al., 2021). Secondly, peer-to-peer information exchange and experience sharing enable students to acquire valuable learning resources and strategies, thereby enhancing their comprehension of course content and academic performance (Wentzel et al., 2021). Furthermore, perceived peer support contributes to the improvement of students’ academic self-efficacy and motivation, fostering an environment conducive to academic success (Altermatt, 2019). Longitudinal research further substantiates the enduring impact of perceived peer support on academic adjustment. A study conducted by Worley et al. (2023) demonstrates that early perceived peer support during the transition from secondary to tertiary education not only effectively predicted academic improvement in higher education but also enhanced academic achievement through the facilitation of positive peer interactions, thereby significantly strengthening students’ academic adjustment. These findings underscore the sustained significance of perceived peer support in students’ adjustment to higher education environments.

Perceived peer support is particularly pronounced in higher vocational education. Among higher vocational college students, over 90% are first-generation college students who encounter multiple challenges, including insufficient academic guidance from their families (Jenkins et al., 2013) and deficiencies in family, cultural, and social capital (Li et al., 2023). In this context, perceived peer support emerges as a critical factor in compensating for the lack of educational resources at home and facilitating academic adjustment. Information sharing and experience exchanging between peers specifically enable higher vocational college students to better comprehend professional knowledge and develop vocational competencies, thereby enhancing their academic adjustment to the learning environment (Lundberg, 2014).

In summary, perceived peer support is a crucial external resource for academic adjustment among higher vocational college students and may play a significant role in compensating for their limited family and social capital. Drawing on the aforementioned theoretical and empirical research, this study proposes the research *hypothesis 1: Perceived peer support positively predicts the academic adjustment of higher vocational college students.*

### 1.2 Mediating effect of academic hope

Snyder (2002) defines hope as a crucial psychological drive and cognitive process that enables individuals to achieve their goals. According to Hope Theory, hope comprises two essential components: pathway thinking and agency thinking. Pathway thinking pertains to an individual’s capacity to devise one or more viable routes to attain their objectives. At the same time, agency thinking reflects the motivation and belief in one’s ability to utilize these routes to reach those goals (Snyder et al., 1991). Academic hope represents the application of Hope Theory in the educational context, encompassing students’ expectations for academic success, and their confidence and motivation to realize these aspirations (Sympson and Snyder, 1997). Specifically, academic hope not only aids students in establishing clear academic objectives (pathway thinking) but also fosters sustained learning motivation (agency thinking) and encourages the development of effective strategies to address academic challenges (Snyder, 2002). In contrast to general hope, academic hope concentrates more on goal setting, strategy formulation, and maintaining motivation within educational settings. Consequently, it correlates strongly with predicting students’ academic achievement and adjusting to academic environments (D’Amico Guthrie and Fruiht, 2020).

The Social Support Theory and the Conservation of Resources (COR) Theory provide a robust theoretical framework for understanding the relationship between perceived peer support, academic hope, and academic adjustment. The central effect hypothesis of Social Support Theory posits that social support can directly enhance an individual’s health and adaptive behavior (Cohen and Wills, 1985). Meanwhile, the COR Theory asserts that individuals cope with external pressures and achieve positive adaptation by acquiring, accumulating, maintaining, and utilizing various resources, including personal abilities, social support, knowledge and material conditions (Hobfoll et al., 2018). When confronted with stress, individuals with more resources are better equipped to navigate challenges and attain favorable adaptation outcomes. Conversely, a scarcity of resources may trap individuals in a detrimental cycle of resource depletion, which further intensifies stress and diminishes adaptability (Hobfoll et al., 2018).

The COR Theory postulates that social support, serving as a fundamental external resource, facilitates the development of positive psychological resources in individuals (Hobfoll et al., 2018).

Among diverse forms of social support, perceived peer support is instrumental in fostering academic hope among students (Kwok et al., 2024). Research by Fu et al. (2019) empirically demonstrates that social support constitutes a pivotal factor in determining levels of hope. This finding extends beyond the context of left-behind children to broader higher education settings. Furthermore, structured peer support initiatives, specifically peer learning workshops, have demonstrated efficacy in enhancing students’ academic engagement and positive attitudes while simultaneously elevating their levels of academic hope (Azadianbojnordi et al., 2020). These findings underscore the distinctive contribution of perceived peer support to developing academic hope. As a critical psychological resource, academic hope enables students to manage academic pressures and enhance their academic adjustment effectively (Hazan-Liran and Miller, 2022; Wong and Yuen, 2023). Specifically, students with higher academic hope demonstrate a greater propensity to establish clear academic objectives, maintain self-efficacy in goal achievement, and exhibit enhanced coping and adaptive capabilities when confronting academic challenges (Snyder et al., 2018). Moreover, during periods of intense academic pressure or setbacks, academic hope facilitates students’ positive adjustment of coping strategies, strengthening their academic adjustment (Hansen et al., 2014). These findings substantiate the fundamental role of academic hope in enhancing students’ academic performance and adaptability.

D’Amico Guthrie and Fruiht (2020) provide compelling empirical evidence for understanding the intricate relationships between perceived peer support, academic hope, and academic adjustment. Their research revealed that supportive peer relationships influence students’ academic success through two pathways. The first pathway represents a direct route, wherein perceived peer support enhances students’ integration with the campus community, directly facilitating academic success. The second pathway operates indirectly; supportive relationships enhance students’ academic hope, promoting academic performance and retention rates (D’Amico Guthrie and Fruiht, 2020). This investigation not only substantiates the direct impact of perceived peer support on academic adjustment but also elucidates the potential mediating role of academic hope in this process, establishing a foundation for constructing an integrated theoretical framework.

Drawing from the COR Theory, perceived peer support functions as a critical external resource that facilitates the development of individuals’ positive psychological resources (Hobfoll et al., 2018). For higher vocational college students specifically, perceived peer support may enhance academic adjustment through multiple mechanisms. Firstly, perceived peer support strengthens higher vocational college students’ confidence in establishing and achieving academic objectives. This enhanced academic hope directly increases their academic engagement and adjustment levels and potentially augments their self-efficacy, thereby promoting superior academic adjustment (Snyder, 2002). Secondly, perceived peer support provides higher vocational college students with both emotional sustenance and academic assistance, mitigating academic stressors and challenges encountered during their educational journey, thus directly enhancing their academic adjustment (Cohen and McKay, 2020). Therefore, academic hope may play an important mediating role in the relationship between perceived peer support and academic adjustment.

Drawing upon the integration of Hope Theory, Social Support Theory, and COR Theory, along with relevant empirical research, *Hypothesis 2* was proposed*: Academic hope acts as a mediator in the relationship between perceived peer support and academic adjustment among higher vocational college students.*

### 1.3 Mediating effect of professional identity

As a crucial psychological resource, professional identity plays a vital role in students’ academic adjustment. It directly influences students’ learning motivation and enhances their adaptability by strengthening their sense of belonging and responsibility toward their chosen field (Chu et al., 2021). Specifically, students with a stronger professional identity typically exhibit greater interest and commitment to their field of study, engage more actively in academic activities, and demonstrate increased persistence and adaptability when confronted with academic challenges (Horrill et al., 2021; Krishna et al., 2023).

During this process, perceived peer support is a vital social resource that is crucial to facilitating. Research indicates that social support, particularly perceived peer support, significantly predicts students’ professional identity (Zhao et al., 2023). Additionally, a quantitative study further demonstrated the significant predictive influence of perceived peer support on developing professional identity among nursing graduate students (Horrill et al., 2021). In essence, perceived peer support enhances students’ sense of belonging and identification with their field of study by providing emotional and academic assistance (Horrill et al., 2021). In professional identity formation, structured peer support mechanisms play an indispensable role. Studies have shown that peer mentoring programs bolster professional identity by aiding students in integrating into the professional community through emotional and academic support (Krishna et al., 2023). As a social resource, perceived peer support can motivate students and enhance their sense of achievement and interest in a specific discipline, thereby improving academic performance and retention rates. Although research has explored the positive impact of perceived peer support on students’ academic adjustment, there remains a notable gap in the literature regarding the role of professional identity as a mediating variable in higher vocational education. Drawing upon the framework of COR Theory, individuals manage external pressures by acquiring, accumulating, and maintaining resources, with social support and psychological resources serving as fundamental elements in stress management (Hobfoll et al., 2018). Professional identity, as a vital psychological resource, may represent a significant pathway through which students translate perceived peer support into improved academic adjustment. Specifically, when higher vocational college students receive peer support, professional identity can act as an essential mediating bridge, facilitating the conversion of emotional and academic support from peers into enhanced learning motivation and adaptability. This mediating process may bolster students’ identification with their professional identity and substantially enhance their academic engagement and performance (Krishna et al., 2023). For example, perceived peer support helps students better integrate into professional communities, enhancing their sense of belonging and responsibility toward their profession, thereby improving their academic adjustment (Chu et al., 2021; Horrill et al., 2021).

In summary, drawing on the integrated perspectives of COR Theory and Social Support Theory, along with relevant empirical research, this study proposes the following *Hypothesis 3: Professional identity serves as a mediator between perceived peer support and academic adjustment among higher vocational college students.*

### 1.4 The chain mediating effect of academic hope and professional identity

According to the COR Theory, individuals strive to accumulate, maintain, and protect valuable resources (Hobfoll et al., 2018). As a crucial psychological resource, academic hope significantly enhances students’ professional identity (Chang et al., 2023; Dong et al., 2020). Specifically, academic hope aims to positively influence professional identity through resource accumulation in the following aspects.

Firstly, academic hope fosters students’ active participation in professional learning activities by stimulating their intrinsic learning motivation (Snyder, 2002). Intrinsic motivation serves as a crucial driver for students’ sustained engagement in their studies, enabling them to remain focused and enthusiastic throughout the learning process while also assisting them in overcoming challenges and difficulties. This sustained active participation not only contributes to students’ academic success but also facilitates the accumulation of professional knowledge and skills, which are essential for developing a professional identity (Azadianbojnordi et al., 2020).

Secondly, academic hope assists students in clarifying their career goals and formulating concrete implementation plans (Snyder et al., 2018). The clarity of career goals provides students with a sense of direction and purpose and bolsters their confidence and expectations regarding future career development (Nicola-Richmond et al., 2023). This clear career path further strengthens students’ sense of belonging to and identification with their majors, as they can closely connect their current learning with their future career trajectories, thereby imbuing their learning with a deeper significance (Zhu et al., 2021). Additionally, the clarity of career goals can inspire students to actively plan and manage their academic resources, fostering a virtuous cycle in their academic and career development.

In summary, academic hope is a crucial psychological resource that enables students to accumulate academic and career-related assets through two primary pathways: fostering intrinsic learning motivation and clarifying career goals. This process significantly enhances their professional identity. Furthermore, it aligns with the fundamental principles of COR Theory, which posits that individuals achieve positive psychological and behavioral outcomes by accumulating and safeguarding resources (Hobfoll et al., 2018). This framework provides both theoretical support and practical insights for understanding the influence of academic hope on students’ professional identity.

Building on this theoretical framework, the present study proposes the following research *Hypothesis 4: Academic hope and professional identity play a chain mediating role between perceived peer support and academic adjustment of higher vocational college students.*

## 2 Materials and methods

### 2.1 Participants

This study focuses on students at the specialized level of higher vocational colleges in China, including those who entered through various pathways such as the single examination and single enrollment, the general college entrance examination, middle and higher vocational connections, and independent enrollment. A multi-stage stratified random sampling method was employed to ensure a representative and diverse sample. In the first stage, based on the “China Development Index 2022” published by Renmin University of China, the country was divided into three categories: developed regions, moderately developed regions, and underdeveloped regions. From these categories, 15 representative provinces encompassing a total of 35 higher vocational colleges were selected, thereby ensuring the sample’s adequacy in terms of geographical distribution and economic development levels. In the second stage, considering that junior students are primarily engaged in off-campus internships, the sampling proportions for each grade were adjusted to focus on freshmen and sophomores, while still retaining a certain proportion of juniors. This approach not only ensures the availability of the sample but also maintains its adequacy across different grades.

Data were collected using paper questionnaires administered collectively by classes and organized by uniformly trained counselors and classroom teachers. A total of 10,000 questionnaires were distributed in this study, resulting in 9,075 valid responses after excluding invalid submissions. The average age of the valid subjects was 19.5 years (*SD* = 1.45), with ages ranging from 17 to 22 years. The effective recall rate was 90.75%. The demographic characteristics of the questionnaire sample are detailed in Table 1.

**Table 1:** Characteristics of higher vocational college student sample (*N* = 9,075).

Features	Category	Number of people	Percentage
Gender	Male	4,524	49.90%
	Female	4,551	50.10%
Grade	Freshman	5,649	62.20%
	Sophomore	2,939	32.40%
	Junior	487	5.40%
Region	Developed regions	4,103	45.20%
	Moderately developed regions	3,617	39.90%
	Less developed regions	1,355	14.90%
Parental education background	First-generation college students	8,178	90.10%
	Non-first-generation college students	896	9.90%
Place of origin	Rural	6,008	66.20%
	Township	1,555	17.10%
	Urban	1,512	16.70%
High school type	Ordinary high school	5,575	61.40%
	Vocational high School	3,500	38.60%
Institution type	Public	5,518	60.80%
	Private	3,557	39.20%

### 2.2 Measures

In this study, we primarily employed four standardized scales: the Academic Adjustment Scale, the Academic Hope Scale, the Perceived Peer Support Scale, and the Professional Identity Scale, specifically designed for higher vocational college students. Additionally, we gathered other significant factors that may influence academic adjustment as control variables. These include whether the students are first-generation college students, their educational background prior to enrollment (regular high school or vocational high school), and their self-assessed achievement levels. These factors were considered control variables because they could influence academic adjustment. For instance, “whether one is a first-generation college student” is used as a control variable, primarily because this identity characteristic may have a potential impact on students’ academic adjustment. First-generation college students, due to the lack of higher education experience support from their families, often face unique challenges in areas such as accessing academic resources, psychological preparedness, and integration into campus life (Jenkins et al., 2013). Additionally, a student’s pre-enrollment educational background, whether from a general or vocational high school, may shape their learning foundations and strategies, potentially impacting academic adjustment. Lastly, the self-assessment of achievement level represents students’ subjective evaluations of their academic performance, which may be closely linked to motivation and learning strategies. Therefore, considering these factors as control variables allows for the exclusion of their confounding effects, thereby facilitating a more accurate understanding of the independent effects of academic hope, peer support, and professional identity on academic adjustment.

The self-assessed achievement levels were based on the research methodology of Ding et al. (2023). Participants were asked to evaluate their academic level according to their overall performance in the class during the last few exams. The evaluation grades were categorized into three intervals, assigned the values of 1 (below average), 2 (average), and 3 (above average). These control variables will be integrated into the model during the data analysis phase to enhance the accuracy and explanatory power of the findings.

#### 2.2.1 The academic adjustment scale for higher vocational college students

Existing academic adjustment assessment tools primarily target the general undergraduate student population and often fail to accurately reflect the academic adjustment characteristics of higher vocational college students. This study developed an Academic Adjustment Scale specifically tailored for this demographic. The scale’s development adhered to a rigorous psychometric procedure.

Initially, a systematic literature review and semi-structured interviews with 42 higher vocational college students were conducted to establish the conceptual framework of the scale. Following this, several rounds of expert discussions led to the creation of an initial scale comprising 61 items. A five-point Likert scale was employed for scoring (1 = very inconsistent, 2 = relatively inconsistent, 3 = generally consistent, 4 = relatively consistent, 5 = very consistent). In the item analysis and exploratory factor analysis phase, the questionnaire was administered to 1,045 students across 10 higher vocational colleges and universities, yielding a valid recovery rate of 87.75% (*N* = 917). After conducting item analysis, 39 items were retained based on indicators such as item decision value, item-total score correlation, corrected item-total score correlation, and changes in the alpha coefficient upon item elimination. Exploratory factor analysis ultimately refined the scale to 19 items, organized into three dimensions: learning engagement (6 items), learning strategies (5 items), and learning motivation (8 items). These three factors accounted for a total of 59.47% of the variance. Finally, in the validation factor analysis stage, a questionnaire survey was conducted with 1,023 students from 13 higher education institutions, achieving an effective recall rate of 86.12% (*N* = 881). The results of the validation factor analysis indicated a good model fit (χ^2^/*df* = 2.67, RMSEA = 0.05, SRMR = 0.05, NFI = 0.95, CFI = 0.97, GFI = 0.94). The internal consistency of the scale was found to be satisfactory, with an overall Cronbach’s alpha coefficient of 0.94 and alpha coefficients for the individual dimensions ranging from 0.84 to 0.90. The validity of the self-administered Academic Adjustment Scale for Higher Vocational College Students was assessed using the Academic Adjustment Scale for College Students developed by Feng et al. (2006) as a benchmark. The correlation coefficient between the total scores of the two scales was 0.72 (*p* < 0.05), indicating that the self-administered scale possesses strong validity.

In this study (*N* = 9,075), we employed a self-administered Academic Adjustment Scale for higher vocational college students consisting of 19 items across three dimensions. The Learning Engagement dimension (6 items) evaluated students’ efforts and engagement in the learning process, exemplified by the statement, “I put a lot of effort into my studies in order to get good grades.” The Learning Strategies dimension (5 items) assessed students’ ability to employ effective learning methods, such as, “I will make a detailed study plan for each stage of learning.” The Motivation dimension (8 items) examined the intrinsic motivation that drives students to learn, illustrated by the statement, “I enjoy learning because it promotes my personal growth.” The overall Cronbach’s alpha coefficient for the scale was 0.95, with alpha coefficients for the dimensions ranging from 0.85 to 0.91. These findings suggest that the scale is a reliable and valid measurement tool for assessing the academic adjustment status of higher education students.

#### 2.2.2 Perceived peer support scale

In this study, the Perceived Peer Support dimension of the Multidimensional Social Support Scale was employed to assess the perceived peer support among higher vocational college students. The scale consists of 27 items, which are categorized into three dimensions based on content: emotional support, instrumental support, and informational support; as well as three dimensions based on source: perceived family support, perceived peer support, and perceived teacher support. Each dimension comprises nine items (Zhang et al., 2021). The scale utilizes a 5-point Likert scale for scoring (1 = very inconsistent, 2 = relatively inconsistent, 3 = generally consistent, 4 = relatively consistent, 5 = very consistent). The internal consistency coefficients for the perceived peer support, family support, and teacher support dimensions ranged from 0.87 to 0.90, indicating an overall scale reliability of 0.96 and strong validity indices. These findings confirm that it is a valid instrument for measuring social support.

All items of the Perceived Social Support Scale were initially measured among higher vocational college students. The reliability and validity of each dimension were analyzed: Perceived Family Support, Perceived Peer Support, and Perceived Teacher Support. The results indicated that the Cronbach’s alpha values for the three dimensions ranged from 0.89 to 0.92. Conversely, the alpha coefficient for the total scale was 0.96, indicating a high degree of reliability. Subsequently, we focused on the content analysis of the perceived peer support dimension to advance the core objectives of this study.

Perceived Peer Support consists of three elements: emotional support, instrumental support, and informational support. Peer emotional support encompasses the understanding, encouragement, and companionship individuals receive during peer interactions (e.g., “Providing comfort and encouragement when needed”). Peer instrumental support focus on the assistance and resources provided by peers, such as money, goods, or services. Peers help individuals address specific challenges or enhance their living conditions (e.g., “Offering contacts or other non-material support when needed”). Peer informational support evaluates information, advice, or guidance to assist individuals in better understanding the issues they encounter and potentially identifying solutions (e.g., “Offering information relevant to your situation”).

In this study, the dimension of perceived peer support was demonstrated to be a good psychometric indicator. Internal consistency tests showed that Cronbach’s alpha coefficients for the dimensions ranged from 0.88 to 0.91, and the alpha coefficient for the overall scale reached 0.95. These results underscore the high degree of reliability inherent in the scale, thereby affirming its suitability for measuring the constructs under investigation.

#### 2.2.3 Academic hope scale

This study employed the Academic Hope Scale (Sympson and Snyder, 1997), derived from the Domain-Specific Hope Scale (Revised), to evaluate students’ pathways thinking and motivational thinking within an academic context. The scale comprises six items categorized into two dimensions: Pathways Thinking (three items) and Motivated Thinking (three items). These dimensions assess students’ capacity to devise strategies for achieving academic goals (e.g., “I can come up with a variety of ways to get good grades”) and their intrinsic motivation to pursue academic success (e.g., “I feel motivated to do well in school”). Responses were measured using a five-point Likert scale (1 = very inconsistent, 2 = relatively inconsistent, 3 = generally consistent, 4 = relatively consistent, 5 = very consistent), with higher scores reflecting greater levels of academic hope. The scale showed strong construct validity in this study, with a Cronbach’s alpha of 0.93 overall and 0.89 for both the pathways and motivation dimensions.

#### 2.2.4 Professional identity scale

This study employed the Professional Identity Scale for College Students, developed by Qin (2009), which comprises 23 items across four dimensions: cognitive identity, affective identity, behavioral identity, and appropriateness identity. Cognitive identity (5 items) assesses students’ fundamental understanding of their majors, as exemplified by the item “I know what qualities my major requires of learners.” Affective identity (8 items) measures students’ emotional disposition toward their majors, illustrated by the item “I am willing to work in a job related to my major.” Behavioral identity (6 items) reflects students’ actual major-related behaviors, such as “I often read books related to my major.” Appropriateness identity (4 items) evaluates the perceived congruence between students’ personal traits and their chosen major, as demonstrated by the item “My personality is a good match for my major.” The scale utilizes a five-point Likert scale (1 = very inconsistent, 2 = relatively inconsistent, 3 = generally consistent, 4 = relatively consistent, 5 = very consistent), with higher scores indicating stronger major identification. In this study, Cronbach’s alpha coefficient was 0.96 overall, while the coefficients for the four dimensions ranged from 0.81 to 0.89, demonstrating high internal consistency.

### 2.3 Research procedure

The data collection lasted from late October 2023 to late December 2023, spanning approximately 2 months. Data collection was conducted using paper-based questionnaires to ensure the standardization of responses and the completeness of the data. The entire data collection process was carried out strictly by the established research plan and ethical requirements. The specific procedures were as follows:

**Step 1.** Questionnaire preparation and distribution. Before formal administration, the research team meticulously designed and printed the questionnaire. Additionally, standardized training was provided to the faculty who were involved in the distribution and administration of the questionnaires. The questionnaires were disseminated on a class-by-class basis, with trained counselors or instructors overseeing the process. Before distribution, students were informed about the anonymity and confidentiality of the questionnaire and the entirely voluntary nature of their participation.
**Step 2.** Questionnaire completion and administration. Students completed the questionnaires collectively in classrooms under supervision. At first, counselors or instructors explained the research background, the confidentiality of the questionnaire, and the voluntary nature of participation briefly. Participants were also informed that they could withdraw at any time without any consequences. The questionnaire administration was conducted under supervision, with students instructed to complete the instruments independently within 20–30 min to ensure response validity while mitigating potential participant fatigue.
**Step 3.** Questionnaire collection and organization. Upon completion of the questionnaires counselors or teachers collect and seal them. Subsequently, these sealed questionnaires are returned to the research team on a school-by-school basis. Stringent confidentiality measures are implemented during both the transportation and storage of the questionnaires to ensure the security and integrity of the data.
**Step 4.** Data cleaning and quality control. The research team conducted multiple rounds of data cleaning and quality control on the collected questionnaires. This process included completeness checks (removing questionnaires with incomplete responses to key questions or significant missing content), logical consistency checks (e.g., verifying the consistency of responses to reverse-coded items and assessing the reasonableness of answers to sequential questions), and compliance reviews (removing questionnaires with random or duplicate responses) to ensure the accuracy and reliability of the data.
**Step 5.** Ethical review and incentive measures. This study has received approval from the Academic Ethics Committee of the School of Psychology at Northeast Normal University (Ethics Approval Number: 2023030) and strictly adheres to the ethical guidelines for psychological research. To encourage students to actively participate in completing the questionnaire, each student who completes it receives a 3-Yuan We Chat red envelope as a reward. Class counselors or teachers manage the distribution of these rewards to ensure fairness and transparency.

### 2.4 Data analyses

In this study, multiple statistical methods were employed to analyze the collected data comprehensively. Initially, descriptive statistical analysis and Pearson correlation analysis were conducted using SPSS 26.0 to establish a preliminary understanding of the relationships among variables. To assess potential common method bias, a Harman’s single-factor test was performed. Subsequently, confirmatory factor analysis was executed using Amos 26.0 software to verify the structural validity of the scales. To examine the mediating effects of academic hope and professional identity, 95% confidence intervals for indirect effects were estimated using the Bootstrap method with 5,000 resamples. Further analyses were conducted using the PROCESS macro (Model 6) in SPSS 26.0 to validate the serial mediation effect.

## 3 Results

### 3.1 Common method variance test

In this study, all variables were measured using self-report scales, which may be susceptible to common method bias. To evaluate the potential impact of this bias, a Harman one-way test was conducted. The results indicated that a total of seven factors had an eigenvalue greater than 1, while the total variance explained by the first principal factor was 35.11%. This value did not exceed the critical threshold of 40%, suggesting that the data were not significantly influenced by common method bias (Zhou and Long, 2004).

### 3.2 Descriptive statistics and correlation analysis of variables

This study conducted correlation analyses to examine the relationships among academic adjustment, perceived peer support, academic hope, professional identity, and demographic variables (high school type, self-assessed achievement level, and parental educational background). The results revealed moderately strong positive correlations between perceived peer support and academic hope, as well as between professional identity and academic adjustment. Self-assessed achievement levels demonstrated significant but weak positive correlations with perceived peer support, academic hope, professional identity, and academic adjustment. High school type (general versus vocational) showed no significant correlation with professional identity or perceived peer support, while exhibiting significant but weak correlations with academic hope and academic adjustment. Similarly, parental educational background (first-generation versus non-first-generation college students) displayed significant but weak positive correlations with perceived peer support, academic hope, professional identity, and academic adjustment.

Overall, perceived peer support, academic hope, and professional identity emerge as the most critical variables influencing academic adjustment, whereas academic performance appears to have a relatively minor impact on academic adjustment. Additionally, factors such as the type of high school attended and parental educational background demonstrate very limited effects on academic adjustment and may lack practical significance. The specific results are shown in Table 2.

**Table 2:** Descriptive statistics and correlation coefficient matrix for each variable (*N* = 9,075).

	M + SD	1	2	3	4	5	6	7
1. AA	3.72 ± 0.59	1						
2. AH	3.82 ± 0.70	0.70**	1					
3. PI	3.91 ± 0.64	0.66**	0.68**	1				
4. PPS	3.72 ± 0.81	0.45**	0.49**	0.46**	1			
5. HST	–	−0.05**	−0.05**	−0.00	0.01	1		
6. SAAL	2.16 ± 0.56	0.27**	0.28**	0.23**	0.12**	−0.14**	1	
7. PEB	–	0.03**	0.03**	0.03**	0.03**	−0.05**	0.04**	1

M, mean; SD, standard deviation; AA, academic adjustment; AH, academic hope; PI, professional identity; PPS, perceived peer support; SAAL, self-assessment achievement levels; HST, high school Type (0 = regular high school, 1 = vocational high school); PEB, parental educational background (0 = first-generation college student, 1 = non-first-generation college student) are dummy variables. Statistical significance is denoted as follows: *** *p* < 0.001, ** *p* < 0.01, * *p* < 0.05 (the same below).

### 3.3 Chain mediating model test

**Figure 2:**
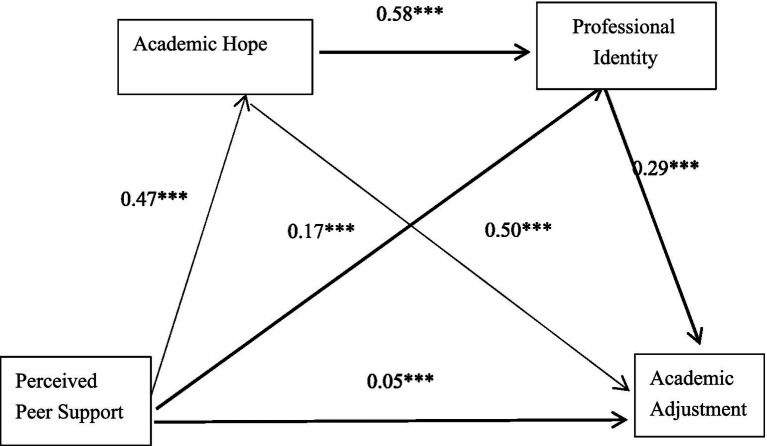
Chain mediating model diagram (*N* = 9,075).

This study investigated the mediating roles of academic hope and professional identity in the relationship between perceived peer support and academic adjustment, utilizing the SPSS macro program PROCESS Model 6 developed by Hayes and Scharkow (2013). After controlling for variables such as the type of high school, self-assessed achievement level, and parents’ educational background, we conducted a series of regression analyses. The results are presented in Tables 3, 4, as well as Figure 2.

**Table 3:** Chain mediating model test of academic hope and professional identity between perceived peer support and academic adjustment (*N* = 9,075).

Regression model		Overall fitting index			Regression coefficient significance			
Dependent variable	Independent variables	*R*	*R* ^2^	*F*	*B*	*β*	*SE*	*T*
AA		0.49	0.24	697.73***				
	Intercept				2.13		0.04	66.75 ***
	PPS				0.30	0.41	0.01	44.16***
	HST				−0.02	−0.02	0.01	−2.11***
	SAAL				0.23	0.21	0.01	22.84***
	PEB				0.01	0.01	0.02	0.74
AH		0.54	0.29	737.12***				
	Intercept				1.75		0.04	48.25***
	PPS				0.40	0.47	0.01	52.49***
	HST				−0.04	−0.03	0.01	−2.83***
	SAAL				0.27	0.22	0.01	24.18***
	PEB				0.02	0.01	0.02	0.87
PI		0.70	0.48	1417.61***				
	Intercept				1.21		0.03	37.54***
	PPS				0.13	0.17	0.01	19.00***
	AH				0.54	0.58	0.01	65.06***
	HST				0.04	0.03	0.01	4.40***
	SAAL				0.06	0.05	0.01	6.63***
	PEB				0.02	0.01	0.01	0.94
AA		0.77	0.59	2170.64***				
	Intercept				0.83		0.03	29.46***
	PPS				0.04	0.05	0.01	6.48***
	AH				0.41	0.50	0.01	51.16***
	PI				0.26	0.29	0.01	30.73***
	HST				−0.02	−0.01	0.01	−1.84***
	SAAL				0.06	0.05	0.01	7.64***
	PEB				0.00	0.00	0.01	−0.05

AA, academic adjustment; AH, academic hope; PI, professional identity; PPS, perceived peer support; SAAL, self-assessment achievement levels; HST, high school type (0 = regular high school, 1 = vocational high school); PEB, parental educational background (0 = first-generation college student, 1 = non-first-generation college student) are dummy variables.

**Table 4:** Total effects, direct effects, and mediating effects in the relationship between perceived peer support and academic adjustment among higher vocational college students (*N* = 9,075).

Pathways	Effect	Boot SE	95% CI		Relative effect quantity
			Boot LLCI	Boot ULCI	
Total effect	0.30	0.02	0.28	0.31	
Mediating effect	0.26	0.01	0.25	0.27	86.67%
PPS → AH → AA	0.17	0.01	0.16	0.18	56.67%
PPS → PI→AA	0.03	0.00	0.02	0.04	10.00%
PPS → AH → PI→AA	0.06	0.00	0.05	0.07	20.00%
Direct effect (PPS → AA)	0.04	0.01	0.03	0.05	13.33%

PPS, perceived peer support; AH, academic hope; AA, academic adjustment; PI, professional identity.

Firstly, perceived peer support demonstrated a significant positive predictive effect on the academic adjustment of higher vocational college students (*β* = 0.41, *SE* = 0.01, *p* < 0.001), confirming research hypothesis 1. After introducing two mediating variables: academic hope and professional identity, the direct effect of perceived peer support on academic adjustment decreased but remained significant (*β* = 0.05, *SE* = 0.01, *p* < 0.001). Secondly, we assessed the mediating effect of academic hope. The results indicated that perceived peer support significantly and positively predicted academic hope (*β* = 0.47, *SE* = 0.01, *p* < 0.001), which, in turn, significantly and positively predicted academic adjustment (*β* = 0.50, SE = 0.01, *p* < 0.001). Using the bias-corrected percentile Bootstrap method (sample size = 5,000), we identified a significant mediating effect of academic hope, with an effect size of 0.17 and a 95% confidence interval of [0.16, 0.18], accounting for 56.67% of the total effect. This finding supported research hypothesis 2. Thirdly, we examined the mediating effect of professional identity. Analyses revealed that perceived peer support significantly and positively predicted professional identity (*β* = 0.17, *SE* = 0.01, *p* < 0.001), which subsequently significantly and positively predicted academic adjustment (*β* = 0.29, SE = 0.01, *p* < 0.001). The mediating effect of professional identity was also significant, with an effect size of 0.03 and a 95% confidence interval of [0.02, 0.04], accounting for 10.00% of the total effect, thereby validating research hypothesis 3. Finally, we investigated the chain mediated effect of academic hope and professional identity. The results demonstrated that academic hope significantly and positively predicted professional identity (*β* = 0.58, *SE* = 0.01, *p* < 0.001), with a significant chain mediated effect size of 0.06 and a 95% confidence interval of [0.05, 0.07], accounting for 20.00% of the total effect, thus supporting research hypothesis 4. Notably, among the three significant indirect effects, academic hope exhibited the largest mediating effect, representing 65.38% (0.17/0.26) of the total indirect effects. This finding underscores the essential role of academic hope in the process through which perceived peer support impacts academic adjustment.

## 4 Discussion

This study aimed to explore the mechanisms of perceived peer support in facilitating the academic adjustment of higher vocational college students, with a particular emphasis on the chain mediating roles of academic hope and professional identity. The findings not only confirmed the direct positive effect of perceived peer support on academic adjustment but also illuminated the mediating roles of academic hope and professional identity. Notably, this study is the first to validate the chain mediating roles of academic hope and professional identity within a higher education context, offering a novel perspective for understanding the complex mechanisms underlying the academic adjustment of higher education students.

### 4.1 The effect of perceived peer support on academic adjustment

The results reveal that perceived peer support significantly affect academic adjustment, even after controlling for variables such as high school type, self-assessed academic performance, and parental educational background. This finding was consistent with previous studies and further extend the applicability of perceived peer support within higher vocational education. Previous studies have shown that peer support can effectively alleviate academic stress, enhance academic self-efficacy, and promote learning adaptation through mechanisms such as emotional support, information sharing, and experience exchange (Camacho-Morles et al., 2021; Wentzel et al., 2021).

Our research findings have been validated in the special context of vocational education, providing additional evidence for these conclusions. However, compared with previous studies that primarily focused on general undergraduate students (Zhang et al., 2021), this study found that peer support has a more significant impact on higher vocational college students.

This may be attributed to the relatively limited family and cultural capital among higher vocational college students (Li et al., 2023), which positions perceived peer support as a crucial resource for adapting to academic and social environments. At the same time, the practice-oriented nature of vocational education may further amplify the significance of peer collaboration. In addition, compared to studies such as Worley et al. (2023), which examine the role of perceived peer support during the transition from high school to university, this study further emphasizes the long-term and multidimensional effects of perceived peer support during the vocational education stage. This study reveals that perceived peer support provides emotional comfort and significantly enhances the academic adjustment of higher vocational college students by establishing social networks and offering learning resources (Lundberg, 2014). This finding presents a novel perspective for research in vocational education.

### 4.2 The mediating effect of academic hope

This study represents a pioneering effort in validating the mediating role of academic hope in the relationship between perceived peer support and academic adjustment within the context of Chinese higher vocational education. The results reveal that the mediating effect of academic hope accounts for 56.67% of the total effect, thereby highlighting its salient role as a mediating variable in the relationship between perceived peer support and the academic adjustment of higher vocational college students. This may be attributed to the deficiencies in family and cultural capital commonly experienced by higher vocational college students (Li et al., 2023), which positions academic hope as a crucial psychological resource in their academic adjustment process. While previous research has confirmed the positive effects of academic hope on academic adjustment (Wong and Yuen, 2023), the current study further elucidates the pathways through which perceived peer support enhances academic adjustment via the specific mediating variable of academic hope. This provides new insights into understanding the resource accumulation process during the academic adjustment of higher vocational college students.

Unlike previous studies that primarily examined students in general colleges and universities (Wong and Yuen, 2023), the present study specifically targets a distinct group of higher vocational college students. These students typically encounter unique challenges, including a weak learning foundation and insufficient motivation. In this context, peer support serves as a vital external social resource, offering higher vocational college students’ emotional comfort and practical assistance and significantly contributing to accumulating and maintaining their academic hope. This is achieved through emotional support, information sharing, and practical help (Cohen and McKay, 2020). According to Hope Theory (Snyder et al., 2018), elevated levels of academic hope facilitate establishing clear academic goals and foster sustained learning. The notable mediating effect of academic hope observed in this study indicates that the influence of perceived peer support on academic adjustment is largely realized through enhancing academic hope, particularly among higher vocational college students, for whom academic hope assumes a particularly salient role as a psychological resource.

### 4.3 The mediating effect of professional identity

The results indicate that professional identity plays a lesser mediating role in this relationship, with only 10.00% of the total effect. This finding not only underscores the importance of professional identity as a psychological resource but also highlights the complexities involved in its development within vocational education. The results were consistent with the studies by Chu et al. (2021) and Horrill et al. (2021), indicating that professional identity can effectively enhance academic adjustment by fostering a sense of belonging and responsibility toward one’s profession among students. However, in contrast to the study conducted by Krishna et al. (2023), the mediating effect identified in this study is relatively small. This discrepancy may reflect the unique characteristics of vocational college students. In contrast to their counterparts in traditional higher education institutions, these students often select their fields of study based more on employment prospects and practical considerations than on personal interest or academic hope. Consequently, this pragmatic orientation may attenuate the role of professional identity in facilitating academic adjustment.

From the perspective of COR Theory, perceived peer support, as an external resource, contributes to the formation of professional identity to a certain extent by enhancing students’ sense of belonging to the professional community (Chen Q. et al., 2023; Chen Y. et al., 2023). Professional identity considered an internal psychological resource, further facilitates the accumulation of resources in the learning process by strengthening students’ sense of professional belonging and value identity, thereby creating what Hobfoll et al. (2018) describes as a “resource acquisition spiral.” This finding aligns with Pifer and Baker (2016) characterization of professional identity as a critical psychological resource. However, the present study identified a relatively small mediating effect of professional identity, which warrants special attention and further investigation. Several factors may account for the limited mediating effect of professional identity: First, as Pifer and Baker (2016) noted, the development of professional identity is a complex and protracted process, particularly within the context of higher education, which may be influenced by various external factors; second, many higher vocational college students enter their programs with uncertainty regarding their choice of major and may lack in-depth knowledge and interest, which can hinder the rapid development of professional identity; finally, the cross-sectional design of the present study may not have adequately captured the dynamic process of professional identity development, especially its long-term impact on academic adjustment. Future research could further investigate the dynamics of professional identity and its extensive effects on academic adjustment through a longitudinal design.

### 4.4 The chain mediating effect of academic hope and professional identity

This study is the first to validate the chain mediating roles of academic hope and professional identity in the relationship between perceived peer support and academic adjustment within the context of vocational education. The findings indicate that perceived peer support enhances the academic hope of higher vocational college students, which subsequently strengthens their professional identity, thereby ultimately facilitating the development of academic adjustment. This discovery enriches the application of COR Theory in vocational education and provides a novel theoretical perspective for understanding the complex mechanisms underlying the academic adjustment of higher vocational college students. The results are consonant with existing studies suggesting that academic hope promotes professional identity (Chang et al., 2023; Dong et al., 2020), thereby extending this theoretical relationship further.

Specifically, within the context of vocational education, this study diverges from previous research that primarily focused on single mediating effects by uncovering the complete resource accumulation pathway from perceived peer support to academic adjustment. This finding supports the “resource gain spiral” and expands its applicability to vocational education.

Firstly, perceived peer support, which encompasses external support resources such as academic guidance and emotional assistance, activates the crucial cognitive resource of academic hope among higher vocational college students (Azadianbojnordi et al., 2020). As a positive psychological resource, academic hope enables students to formulate clear academic goals and equips them with the motivation and perceived pathways to achieve these goals.

Secondly, academic hope aims to promote the development of professional identity among higher vocational college students through a dual pathway. On the one hand, academic hope enhances students’ sense of professional competence and belonging by improving their learning engagement and academic achievements. On the other hand, academic hope provides meaningful support for developing professional identity by bridging current academic goals with future career aspirations. This finding resonates with the discussion by Diemer and Blustein (2007) on the intimate relationship between career aspirations and professional identity.

Furthermore, professional identity enhances academic adjustment through an integrative mechanism involving cognition, affection, and behavior. This process manifests as a positive dynamic cycle of resource enhancement. The strengthening of professional identity increases students’ recognition of the value of professional learning at the cognitive level and deepens their emotional engagement at the affective level, which subsequently catalyzes proactive learning behaviors at the behavioral level. These positive learning behaviors, in turn, reinforce professional identity, thereby creating a continuous improvement in academic adjustment. This finding not only supports the argument put forth by Tan et al. (2017) that professional identity facilitates academic adjustment but also elucidates the specific mechanisms involved in this process.

This study verifies the chain mediating effect of academic hope and professional identity, uncovering the resource accumulation pathway for academic adjustment among higher vocational college students. The findings suggest that perceived peer support exerts a direct influence on academic adjustment and establishes a dynamic process of resource transformation and accumulation through the interactive effects of academic hope and professional identity. This provides a new theoretical framework for future research and offers significant insights into vocational education practices. Specifically, by strengthening perceived peer support, enhancing academic hope, and fostering professional identity, the academic adjustment and holistic development of higher vocational college students can be effectively promoted.

## 5 Research contributions

Firstly, the study comprehensively integrate the Social Support Theory and the COR Theory to elucidate the academic adjustment process of higher vocational college students. This integration highlights perceived peer support as a vital external resource, while identifying academic hope and professional identity as significant internal psychological resources, thereby illuminating their interactions within the higher vocational education context. Furthermore, this synthesis not only broadens the application of Social Support Theory in the realm of resource accumulation but also enhances the understanding of COR Theory within educational psychology. It offers a novel perspective for comprehending the utilization and transformation of academic adjustment resources among higher vocational college students.

Secondly, this study illuminates the dynamic spiral process of resource acquisition within the framework of COR Theory (Hobfoll et al., 2018). The identified chain mediating effects—where perceived peer support enhances academic hope, which subsequently fosters professional identity and improves academic adjustment—demonstrate a dynamic process of resource accumulation and transformation. This finding illustrates how external support facilitates the accumulation of internal resources, while the enhancement of these internal resources further strengthens adaptive capacity, creating a positive feedback loop. This dynamic process not only provides a theoretical foundation for understanding the long-term development of academic adjustment among higher vocational college students but also enriches our comprehension of psychological resource mechanisms.

Lastly, this study proposes and validates a multidimensional mediating mechanism model for academic adjustment among higher vocational college students. This model incorporates two interrelated psychological resources: academic hope and professional identity. Within the higher vocational education context, academic hope represents students’ confidence in their academic capabilities and future aspirations (Snyder, 2002), while professional identity reflects students’ recognition of and commitment to their chosen profession (Pifer and Baker, 2016). The interplay between these resources demonstrates how higher vocational college students can enhance their academic adjustment through the synergistic effects of diverse psychological resources when confronting academic challenges.

## 6 Limitations

While this study yielded valuable findings, it is important to acknowledge several limitations.

Firstly, the cross-sectional design employed restricts our ability to draw causal inferences between the variables examined. Future research might benefit from adopting a longitudinal design or experimental approach to elucidate the causal relationships among these variables more effectively.

Secondly, this study’s focus was limited to Chinese higher vocational college students; thus, future investigations could extend to other cultural contexts or educational systems to assess the cross-cultural applicability of these findings.

Third, the present study primarily relied on self-assessment scales to investigate the relationships among perceived peer support, academic hope, professional identity, and academic adjustment, which presents certain limitations. Future studies could enhance understanding by incorporating a diverse range of indicators, including evaluations from teachers and parents, as well as students’ actual academic performance, to provide a more comprehensive view of the academic adjustment process.

## 7 Conclusion

This cross-sectional study found that perceived peer support not only directly predicted the academic adjustment of higher vocational college students, but also indirectly through the independent mediating effects of academic hope and professional identity, as well as the chain mediating effect of academic hope to professional identity. These findings provided new theoretical insights into the complex mechanisms of academic adjustment for higher vocational students and offered valuable guidance for educational practice and policy development.

## Data Availability

The original contributions presented in the study are included in the article/supplementary material, further inquiries can be directed to the corresponding author.
